# Prostatic Therapeutic Efficacy of LENILUTS^®^, a Novel Formulation with Multi-Active Principles

**DOI:** 10.3390/pharmaceutics14091866

**Published:** 2022-09-05

**Authors:** Erik Tedesco, Federico Benetti, Simone Castelli, Andrea Fratter

**Affiliations:** 1 ECSIN-European Center for the Sustainable Impact of Nanotechnology, ECAMRICERT SRL, Corso Stati Uniti 4, 35127 Padova, PD, Italy; 2 INPHA DUEMILA S.r.l., Via Cardinal Ferrari 6, 22066 Mariano Comense, CO, Italy; 3 Department of Pharmaceutical and Pharmacological Sciences, University of Padova, 35131 Padova, PD, Italy; 4 Italian Society of Nutraceutical Formulators (SIFNut), 31033 Castelfranco Veneto, TV, Italy

**Keywords:** urology, BPH, LUTs, LNCaP, prostate, dietary supplements, delivery system

## Abstract

Lower Urinary Tract Symptoms (LUTs) in men are usually associated to benign prostatic hyperplasia (BPH), a non-malignant prostate enlargement. Unfortunately, BPH etiology is still unclear. Recent works highlighted a relevant inflammation role in BPH onset and development. Consequently, to complement the 5-α reductase (and α-adrenergic receptor agonists-based therapy, an anti-inflammatory therapy should be devised. To reduce potential adverse effects of multi-drug treatment, plant extract-based therapies are becoming increasingly common. Serenoa repens, the main phytotherapic treatment for BPH, is not sufficient to front the multi-faceted etiology of BPH. In response to this, a novel, multiple phytotherapic agents-based formulation, LENILUTS^®^, was developed. In the present work, we compared, using an in vitro approach, the prostatic safety and efficacy of LENILUTS^®^ with a commercial formulation, based only on Serenoa repens, and a 5αR inhibitor, Dutasteride. Furthermore, preliminary in vitro experiments to investigate the active principles, bioaccessibility and bioavailability of LENILUTS^®^ were performed. Our results showed a better prostatic safety and therapeutic efficacy of LENILUTS^®^ compared to the commercial formulation and Dutasteride, with increased anti-inflammatory, and pro-apoptotic activity, and a stronger inhibitory effect on the release of the key enzyme 5αR and Prostatic-Specific Antigen (PSA). The limited bioaccessibility and bioavailability of the active principles of LENILUTS^®^ were highlighted. Considering the results obtained, the LENILUTS^®^ formulation is more promising for BPH and LUTs therapy compared to formulations based on Serenoa repens only, but further efforts should be made to improve the bioaccessibility and bioavailability of the active principles.

## 1. Introduction

Lower Urinary Tract Symptoms (LUTS) are a group of urinary symptoms triggered by an obstruction, abnormality, infection or irritation of the lower urinary tract (i.e., the urethra, bladder, bladder neck, urinary sphincter and/or prostate (in men)), and negatively affect the lifestyle of aging men [1]. LUTS can be categorized as being related to either urine storage (urinary frequency, urinary urgency, etc.) or voiding (obstruction) (hesitancy, weak or intermittent stream, etc.), and present as various voiding dysfunctions [2]. While both men and women can be affected, LUTS is most often diagnosed in men affected by a benign enlargement of the prostate, known as Benign Prostatic Hyperplasia (BPH). BPH is an enlargement of the prostate gland, typically in the central zone, which is the zone of the prostate surrounding the urethra. This enlargement, in turn, puts pressure on urethra, increasing outlet resistance, and as a consequence leading to LUTS [3]. At present, it is generally agreed that BPH is a consequence of an androgen receptor-mediated cell proliferation [4], in particular of its smooth muscle component, contraction of which is responsible for many BPH-related symptoms, such as LUTS [5,6]. However, as pointed out in the Medical Therapy of Prostatic Symptoms study [7], a prominent role for inflammation in the insurgence and development of BPH has been proposed [8,9]. Taken together, this evidence seems to suggest a complex etiology for BPH, treatment of which should require a multidrug-based approach. Accordingly, BPH-related LUTS is presently treated with a combination of 5αR inhibitors, which block conversion of testosterone to dihydrotestosterone, α-adrenergic receptor agonists, which favor smooth muscle relaxation, and plant extracts or phytotherapeutic agents [10,11], which can adjuvate and amplify the aforementioned activities. Indeed, formulations based on plant extracts are by far the most popular approach used in the medical management of BPH-induced LUTS [12,13]. Among them, Serenoa repens, extracted from saw palmetto tree berries, is the most popular one [14,15,16]. However, considering its multi-faceted origin [17,18], a formulation based on multiple active principles would likely be preferable in BPH-mediated LUTS treatment. To test this hypothesis, a novel formulation, LENILUTS^®^, in which anti-inflammatory (beta-sitosterol, BS [19,20], Curcuma longa CL [21,22] and oligomeric proanthocyanidins, OPC [23,24]) and anti-oxidant and antibacterial (BS [25,26], CL [27,28] and OPC [29,30]) active principles are blended, was compared to a commercially available Serenoa repens oil mono-component-based formulation. The effect of LENILUTS^®^ and the commercial formulation on different prostatic parameters such as inflammation, 5αR inhibitory activity, prostate-specific antigen (PSA) release and smooth muscle activity were investigated using an in vitro approach, based on an in vitro prostate model. Finally, preliminary in vitro tests, based on an integrated system composed of an in vitro human digestive and an intestinal epithelium model, were performed to determine the bioaccessibility and bioavailability of LENILUTS^®^.

## 2. Materials and Methods

### 2.1. Materials

High-glucose Dulbecco’s Modified Eagle Medium (DMEM), Roswell Park Memorial Institute (RPMI) 1640 Medium, Hanks’ Balanced Salt Saline (HBSS), non-essential amino acids (NEAA), L-glutamine, Penicillin–Streptomycin mix, lipopolysaccharide (LPS), Diclofenac, dihydrotestosterone (DHT), staurosporine (STS) and phorbol 12-myristate 13-acetate (PMA) were purchased from Sigma-Aldrich (St Louis, MO, USA). LNCaP androgen-sensitive human prostate adenocarcinoma cell line (ATCC^®^ CRL-1740™), Caco-2 human colorectal adenocarcinoma cells (ATCC^®^ HTB-37™) and THP-1 (ATCC^®^ TIB-202™) were purchased from ATCC (Manassas, VA, USA). CellTiter 96^®^ AQueous One Solution Cell Proliferation Assay (MTS) and Apo-ONE^®^ Homogeneous Caspase-3/7 Assay were purchased from Promega (Madison, WI, USA). Oxygen Radical Antioxidant Capacity (ORAC) Assay kit was purchased from Cell Biolabs (San Diego, CA, USA). Transwell^®^ inserts were purchased from Millipore (Burlington, MA, USA). Fetal bovine serum (FBS) was purchased from Euroclone (Milan, Italy). Interleukin 1β (IL-1β), Tumor Necrosis Factor α (TNF-α), PSA and DHT ELISA kit were purchased from R&D Systems, PeproTech (London, UK), Abcam (Cambridge, UK), and Cloud Clone (Katy, TX, USA), respectively.

### 2.2. Formulation Composition

An evaluation of the comparative efficacy on prostate and smooth muscle in vitro models was performed for LENILUTS^®^ and a commercially available formulation (CF), the compositions of which are detailed in Supplementary Materials (Table S1), and Dutasteride. Both of formulations were resuspended in dimethyl sulfoxide (DMSO).

### 2.3. Methods

#### 2.3.1. Cell Cultures

##### LNCaP Cell Culture

The LNCaP cells (human prostate cancer line) were kept at 37 °C in a humidified atmosphere with 5% CO_2_ in complete cell culture medium (RPMI-1640 medium supplemented with 10% FBS and 1% Penicillin–Streptomycin mix) from passage 25 to 40. For propagation, cells were subcultivated by trypsinization every 7 d when 80–90% confluent, seeded at a density of 1 × 10^4^ cell/cm^2^, and the medium was changed every other day. LNCaP cells were seeded at a density of 1 × 10^5^ cells/cm^2^ in 96- and 6-well plates for vitality and anti-inflammatory experiments and allowed to adhere for 2 days prior to experiments. A lower cell density (5 × 10^4^ cells/cm^2^) was used for prostate-specific antigen (PSA) experiments, performed in 24-well plates.

##### THP-1 Cell Culture

Human THP-1 monocytes were cultured at a density of 5 × 10^5^ cells/mL and maintained in cell culture complete medium (RPMI-1640 medium with glutamate supplemented with 10% FBS, 100 U/mL penicillin and 100 μg/mL streptomycin) in 5% CO_2_ humidified atmosphere. Cells were subcultured twice a week. A concentration of 500 nM of phorbol myristate acetate (PMA; Sigma-Aldrich, MO, USA) was applied for 24 h to induce macrophage differentiation. At the end of the exposure, the differentiation inducing-medium was replaced with complete cell culture medium and cells cultured for an additional 24 h. Conditioned medium was prepared by seeding 6 × 10^6^ cells in 75 cm^2^ flask, followed by macrophage differentiation and treatment with 1 ng/mL LPS for 6 h. At the end of the LPS treatment, medium was recovered and stored at −80 °C until use.

##### Caco-2 Cell Culture

The Caco-2 cells (human colon adenocarcinoma cells) were seeded in an adhesion flask in cell complete medium (high glucose DMEM, 10% heat inactivated FBS, 1% Non-Essential Amino Acids, 4 mM L-glutamine and 1% penicillin/streptomycin mix) at a density of 2 × 10^3^ cell/cm^2^ in and kept at 37 °C and 5% CO_2_ in a humidified incubator (passage 32 to 42). Cells were subcultivated by trypsinization every 7 d when 80–90% confluent and seeded at a density of 2000 cells/cm^2^. The medium was refreshed every other day.

#### 2.3.2. Evaluation of LENILUTS^®^ Antioxidant Activity

The antioxidant activity of LENILUTS^®^ was evaluated using the ORAC test, following the indications provided on a commercial kit (Cell Biolabs). Briefly, the ORAC test for LENILUTS^®^ was conducted in accordance with the protocol contemplated for food samples, considering both the hydrosoluble and liposoluble components of the formula. In brief, a LENILUTS^®^ tablet was crushed to a powder and the formula was weighed and resuspended in water. After centrifugation to precipitate the fraction not dissolved in the water, the supernatant composed of the water-soluble fraction (hydrophilic fraction) was removed. The pellet was once again resuspended in water, and, after centrifugation, the supernatant collected was added to the hydrophilic fraction. The pellet remaining after the aforementioned steps was resuspended in absolute acetone and shaken at room temperature for 30 min. After centrifugation, the supernatant (lipophilic fraction) was removed. The hydrophilic and lipophilic fractions were adequately diluted in the reaction buffer, and 25 µL of solution was used in each reaction to determine antioxidant activity. After a 30 min pre-incubation phase at 37 °C, a free radical initiator is added, and the reaction is incubated at 37 °C for 60 min, during which fluorescence is monitored at one-minute intervals, using excitation and emission wavelengths of 480 and 520 nm, respectively. For both hydrophilic and lipophilic fraction, the final result was calculated and expressed as μmol Trolox equivalents per gram of sample. Finally, the ORAC value results were determined from the sum of the ORAC values obtained from the hydrophilic and lipophilic fractions.

#### 2.3.3. Evaluation of the Impact of Tested Formulations, Dutasteride and Diclofenac, on LNCaP Cells

To evaluate the impact on human prostatic cellular model of LENILUTS^®^, CF, Dutasteride and the anti-inflammatory drug Diclofenac, and to determine their highest non-toxic concentration, a dose–response curve experiment was performed on LNCaP cells. Briefly, LNCaP cells were treated with increasing concentrations of LENILUTS^®^ (from 0 to 2000 µg/mL), CF (from 0 to 4000 µg/mL) and Dutasteride (from 0 to 372.5 µg/mL) for 6 and 24 h. Diclofenac treatment (from 0 to 1591 µg/mL) was limited to 6 h, corresponding to the duration of anti-inflammatory activity experiments. At the end of incubation time, the vitality of the LNCaP cells was determined by MTS assay, according to the manufacturer’s instructions. The obtained dose–response curves were fitted with OriginLab software (Version 95E, OriginLab Corporation, Northampton, MA, USA) and half-maximal effective concentration (EC50) calculated.

#### 2.3.4. Prostate-Specific Anti-Inflammatory Activity

The prostate-specific anti-inflammatory activity of the tested formulations and drugs was evaluated in a LNCaP cell-based prostatic epithelium in vitro model, with a two-step protocol: (i) 2 h pre-treatment of the prostatic epithelium in vitro model with the highest non-toxic concentrations of formulations and drugs; and (ii) a 4 h exposure to inflammatory stimulus in presence of formulations and drugs. In vitro prostate model inflammation was achieved with the method described by Carmen and colleagues [31]. At the end of the treatment, LNCaP cells were washed with DPBS, scraped in ice-cold PBS, centrifugated, and lysed by sonication in lysis buffer. Following centrifugation at 10,000× *g* for 15 min, the level of interleukin-1beta (IL-1β) and tumor necrosis factor-alpha (TNF-α) cytokines in the obtained supernatants were quantified by commercial ELISA (Enzyme-Linked Immunosorbent Assay) kits, following the manufacturer’s instructions.

#### 2.3.5. LENILUTS^®^ Pro-Apoptotic Activity

The pro-apoptotic activity of LENILUTS^®^ was evaluated with a commercial fluorimetric assay, based on caspase 3/7 activation. The activated caspases selectively cleave a specific substrate, making it fluorescent (Ex: 499 nm, Em: 521 nm), linking the intensity of the produced fluorescence to the cell apoptotic process activation. To investigate the correlation between the anti-inflammatory and pro-apoptotic activity, the same experimental setup described above for the anti-inflammatory activity was applied. The experiments were conducted following the manufacturer’s instructions. STS, a cell death inducer, was used as positive control for apoptosis at 1 μM.

#### 2.3.6. 5α-R Activity

The formulations’ impact on 5αR activity was assessed following the protocol described by Assinder [32]. Briefly, equal amounts of the same LNCaP cell lysate (i.e., same total protein content) were incubated for 16 h at 37 °C under agitation with LENILUTS^®^ (750 μg/mL) and CF (100 µg/mL), in the presence of 5αR cofactor NADPH (100 µM) and the substrate testosterone (100 µM). Dutasteride was used as a positive control (93.1 µg/mL), while no testosterone-incubated cell lysate was considered as negative control. At the end of the incubation period, the reactions were blocked with ice and the DHT content of the different lysate measured by ELISA assay, according to the manufacturer’s indications.

#### 2.3.7. Measurement of PSA Secretion by LNCaP Prostatic Cells

The effect of LENILUTS^®^, CF and Dutasteride on PSA secretion was evaluated in LNCaP prostatic cells, following the protocol described by Kampa and colleagues [33], in the presence and absence of DHT (10 nM), an androgen known to increase PSA release. Secreted PSA levels were measured with a commercial ELISA kit, following the manufacturer’s instructions. The results are expressed as percentage of secreted PSA in cells treated with different formulations compared to control.

#### 2.3.8. In Vitro Digestion Process

A single dose of each formulation listed in Table S1 was digested with an in vitro digestion system composed of three compartments (i.e., oral, gastric, and intestinal compartment) and simulating the physiological human digestion. Briefly, the formulations were incubated in saliva-simulating fluid at 37 ± 1 °C for 5 min, rotating head-over-heels at 55 rpm to simulate the peristaltic movements. Then, gastric juice-simulating fluid (pH 1.3 ± 0.1) was added to the mixture and the pH adjusted to 2.5 ± 0.5 with NaOH (1 M) or HCl (37%). As for the oral compartment, the digesta was maintained under head-over-heels rotation at 37 °C for 2 h. Subsequently, duodenal juice (pH 8.1 ± 0.1), bile (pH 8.2 ± 0.1) and sodium bicarbonate were added, and the pH adjusted to 6.5 ± 0.5. Head-over-heels rotation was maintained for another 2 h. For simulated digestive fluids composition refer to [34]. Once the digestion process was completed, the bioaccessibility of beta-sitosterol and oligomeric proanthocyanidins (OPAs) was determined by high-pressure liquid chromatography (HPLC), while curcumin was quantified with a spectrophotometric approach.

#### 2.3.9. Curcumin Determination

Curcumin was determined spectrophotometrically. Briefly, when resuspended in the organic solvent dimethyl sulfoxide (DMSO), curcumin shows an absorption peak at 420–430 nm (Figure S1A), while, when excited at 420 nm, curcumin produces an emission peak at between 520 and 550 nm (Figure S1B). Considering its peculiar spectral properties, the concentration of curcumin was determined by exciting at 420 nm and measuring the fluorescence intensity at 545 nm. The resulting values were interpolated with a linear calibration curve, obtained with different concentrations of a curcumin standard. This approach ensures a lower limit of detection (LOD) compared to the standard HPLC approach (50 ng/mL compared to 0.05 mg/mL) and allows for the determination of curcumin in the intestinal epithelium.

#### 2.3.10. In Vitro Model of Human Intestinal Epithelium

Absorption and bioavailability of beta-sitosterol, curcuminoids and OPAs were determined using an in vitro model of human intestinal epithelium based on Caco-2 cells. Briefly, Caco-2 cells were seeded at a density of 1.5 × 10^5^ cells/cm^2^ on 1 µm pore size Transwell^®^ polytetrafluoroethylene inserts and left to mature and differentiate for 21 days. In this peculiar environment, characterized by the compartmentalization typical of the Transwell^®^ system, Caco-2 cells acquire the morpho-functional features of the mature enterocyte (presence of microvilli, tight junctions and P-glycoprotein). Absorption experiments were performed between 21 and 28 days post seeding.

#### 2.3.11. Digested Formulations’ Impact on the Viability of the Intestinal Epithelium

The impact of the digested formulations on the viability of the intestinal epithelium was evaluated by adding serially diluted digested formulations in digestive fluids in the apical compartment and incubating for 3 h. In the basolateral compartment, HBSS was added, and digestive fluids (without formulations) were added to the apical side as a negative control. At the end of the exposure period, monolayers were washed with pre-warmed HBSS, and the viability of the intestinal epithelium was evaluated with MTS assay, according to manufacturer’s instructions. The absorbance at 490 nm was determined with a micro-plate reader (Synergy4, Biotek Instruments, Inc., Winooski, VT, USA) and cell viability (%) was expressed as the ratio of the absorbance in the treated groups to that in the control (untreated) group. Bioavailability experiments were performed for the active principles using non-toxic concentrations determined by dose–response curves.

#### 2.3.12. Evaluation of the Bioavailability of Beta-Sitosterol, Curcumin and OPA 

Based on the dose–response curve information and their posology, the digested formulations were added to the apical side of the in vitro intestinal epithelium, while HBSS buffer supplemented with 1% BSA was placed in the basolateral compartment. Due to the lipophilicity of formulation active components, 1% BSA was added to the basolateral compartment for improving their bioavailability. According to the literature [35], the addition of BSA improves the correlation between absorption occurring in Caco-2 cell monolayer and humans. In vitro intestinal epithelia were exposed to the digested formulations containing 12.1 mg/mL of LENILUTS^®^ solution for 1 and 3 h, and beta-sitosterol, curcuminoids and OPCs were measured in both apical (lumen) and baso-lateral (serosal) compartments by HPLC and the spectrophotometric approach, respectively. Bioavailability was then calculated and is expressed as percentage of absorption (%) compared to the amount of the active principles initially loaded in the apical (i.e., luminal) compartment, and concentration (ng/mL), derived from three independent experiments.

#### 2.3.13. Evaluation of Post-Intestinal Absorption Caco-2 Monolayer Barrier Integrity and Viability

After exposure to the digested formulations, the viability and barrier integrity of the Caco-2 monolayer were evaluated. Briefly, once the incubation with the digested formulations was complete, the Caco-2 monolayer was washed and equilibrated in pre-warmed HBSS for 30 min. Then, Caco-2 monolayer barrier integrity was evaluated with an ERS2 Voltohmmeter (Millipore), equipped with a chopstick electrode, by measuring the trans-epithelial electrical resistance (TEER). TEER values are the average of three measurements taken at different points in the well in order to have information as representative of the monolayer as possible. Lucifer Yellow, a fluorescent polar tracer unable to pass through intact tight junctions, was used to assess the paracellular permeability of Caco-2 monolayers. Paracellular permeability was measured by adding 100 µg/mL LY in HBSS in the apical compartment and 1.5 mL of HBSS in the basolateral compartment. Then, after 1 h incubation, the basolateral fractions were collected, and their fluorescence measured with a spectrofluorometer (Synergy 4, Biotek).

The following formula was used to calculate the apparent permeability coefficient (***Papp***, cm/s):

***Papp*** = (Δ*C*
*V*)/(Δ*t*
*A*
*C*_0_)

where Δ*C*/Δ*t* is the flow of the molecule being transported across the monolayer during the incubation time (mM/s), *V* is the volume of the basolateral compartment (cm^3^), *A* is the area of the membrane (cm^2^), *C*_0_ is the initial concentration of the molecule in the apical compartment. Finally, cell viability was evaluated by using MTS assay as described above.

#### 2.3.14. Statistical Analysis

Experiments were performed in triplicate, and the results are presented as average ± standard deviation. Results were statistically analyzed by *t*-test or one-way ANOVA test in case of 3 or more experimental groups, using OriginLab software (OriginLab Corporation, Northampton, MA, USA) and a *p* value of ≤0.05 was considered significant.

## 3. Results

### 3.1. Antioxidant Activity

The antioxidant action of the LENILUTS^®^ was determined using an ORAC test. Table 1 shows the hydrophilic, lipophilic, and total ORAC values, expressed as µmol TE/g of formula ± standard deviation (SD). The results show that the formula possesses an antioxidant action, mainly due to its liposoluble components.

### 3.2. Impact of LENILUTS^®^, CF, Dutasteride and Diclofenac on the In Vitro Prostate model

**Figure 1:**
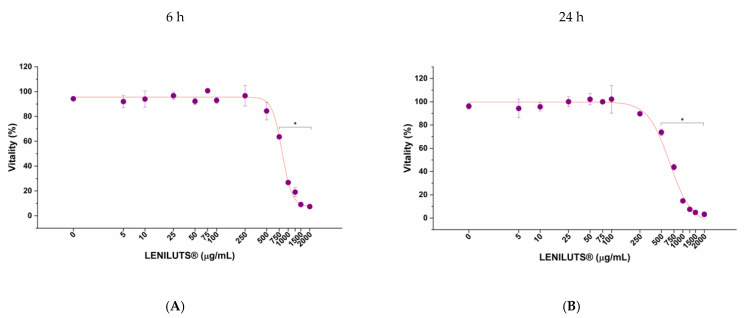

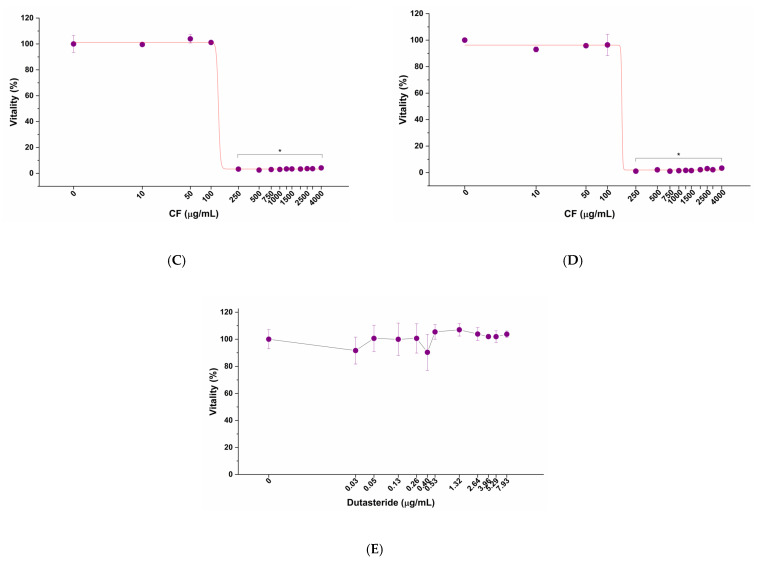
Impact of LENILUTS^®^ (**A**,**B**), CF (**C**,**D**) and Dutasteride (**E**) on in vitro prostate model vitality, following 6 and 24 h exposure. * *p* < 0.05.

Before comparing the efficacy of LENILUTS^®^ with its commercial competitor CF and Dutasteride, we investigated their safety on an in vitro prostate model through dose–response toxicological analysis, considering 6 and 24 h as relevant exposure times. In vitro prostate model vitality was significantly reduced following 6 and 24 h treatment at 750 µg/mL and 500 µg/mL of LENILUTS^®^, respectively (Figure 1A,B), while CF completely abrogated the prostate model vitality at 250 µg/mL, independently of exposure time (Figure 1C,D). With respect to Dutasteride, no adverse effects on the LNCaP-based in vitro prostate model were observed following 24 h at all tested concentrations. (Figure 1E). However, since there are known cases in the literature of the depositing of crystals with possible toxic effects for the cells at concentrations higher than 5.29 μg/mL, this maximum concentration was used for the subsequent assays on the in vitro prostate model. Finally, since Diclofenac is used as a positive control in the inflammation experiment, its impact on in vitro prostate model was also evaluated (Figure S2). EC50 values are reported in Table S2. Based on cytotoxicity results, 6 h exposure–efficacy tests were performed with 500 µg/mL of LENILUTS^®^, 100 µg/mL of CF, 5.3 µg/mL of Dutasteride and 32 µg/mL of Diclofenac, while for 24 h exposure–efficacy, concentrations of 250 µg/mL, 100 µg/mL and 5.3 µg/mL were applied for LENILUTS^®^, CF and Dutasteride, respectively. To further investigate its efficacy, lower concentrations of LENILUTS^®^ were considered (250 µg/mL and 100 µg/mL at 6 and 24-h, respectively).

### 3.3. Effect of LENILUTS^®^, CF and Dutasteride Treatment on Pro-Inflammatory Cytokine Release from the In Vitro Prostate model

**Figure 2:**
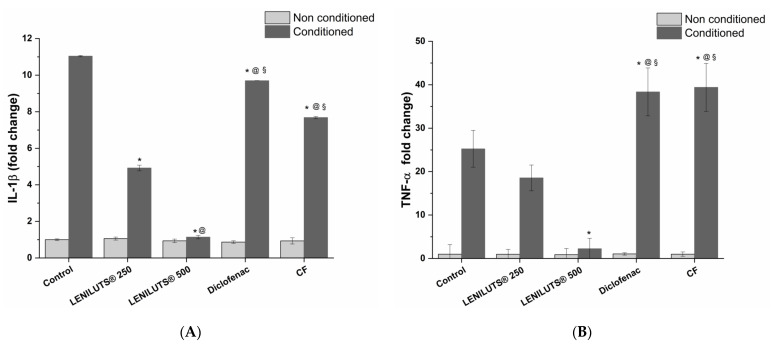
IL-1β (**A**) and TNF-α (**B**) release variation in inflamed LNCaP-based in vitro prostate model before and after treatment with LENILUTS^®^, CF and Diclofenac (positive control). compared to control (Ctrl; untreated LNCaP cells). * *p* < 0.05 vs. control, @ *p* < 0.05 vs. LENILUTS 250, § *p* < 0.05 vs. LENILUTS 500.

In recent years, a direct correlation between inflammation and BPH development has been highlighted [8,9,10]. The anti-inflammatory activity of LENILUTS^®^, CF and Dutasteride was assessed by measuring pro-inflammatory cytokine (IL-1β and TNF-α) release. Diclofenac, an anti-inflammatory drug, was used as positive control. As shown in Figure 2A and Table S3, a significant reduction in IL-1β release compared to the inflamed and untreated in vitro prostate model (11.0 ± 0.0-fold change) was observed for LENILUTS^®^ at both tested concentrations (about 5- and 1-fold changes at 250 and 500 µg/mL, respectively), CF (7.7 ± 0.1 fold change) and Diclofenac (9.7 ± 0.1-fold change) following 6 h treatment. LENILUTS^®^-induced IL-1β release reduction was significantly higher compared to CF, Dutasteride and Diclofenac, with the latter being the least effective. The anti-inflammatory activity of LENILUTS^®^ was further confirmed by TNF-α release (Figure 2B and Table S3). Indeed, in contrast to CF and Diclofenac, LENILUTS^®^, compared to the inflamed, untreated control, demonstrated a decrease in TNF-α release by 30 and 90% at 250 and 500 µg/mL, respectively. The higher LENILUTS^®^ anti-inflammatory activity compared to CF was probably due to the synergistic effect of the phytotherapeutic agents contained in the formulation, such as Pinus spp. [19,20,23,24] and Curcuma longa [36,37].

### 3.4. Pro-Apoptotic Activity of LENILUTS^®^, CF and Dutasteride

As detailed in the previous paragraph, BPH is the most common cause of the development of LUTS. This prostate enlargement is mainly caused by the uncontrolled proliferation of prostatic cells. As a consequence, a formulation that is able to contain such proliferation, via cell death mechanisms like apoptosis and/or necrosis, may slow down the onset and development of BPH. Considering the underlying inflammatory processes, it is also fundamental for such a formulation to affect cell proliferation in an inflamed environment. To this end, we investigated the ability of LENILUTS^®^, CF and Finasteride to induce apoptosis and necrosis in an in vitro prostate model under both non-inflamed and inflamed conditions prostate model. Furthermore, to investigate the possible correlation between anti-inflammatory activity and induction of cell death, the same inflammation protocol was maintained. The activation of key enzymes in the apoptotic signaling cascade (caspase 3 and 7) was used to assess pro-apoptotic activity induction by considered formulations and drugs. 

**Figure 3:**
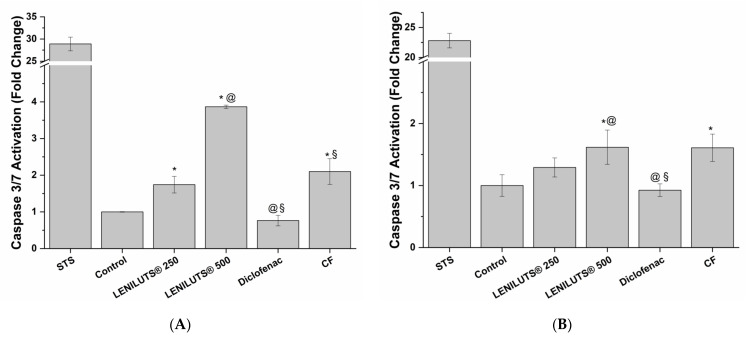
Caspase 3/7 activation compared to non-inflamed, untreated LNCaP cells (**A**, Ctrl) and inflamed, untreated LNCaP cells (**B**, Ctrl) following 6 h treatment with LENILUTS^®^, CF, Finasteride and staurosporine (STS, positive control). * *p* < 0.05 vs. control, @ *p* < 0.05 vs. LENILUTS 250, § *p* < 0.05 vs. LENILUTS 500.

LNCaP prostate cells treated with 250 and 500 µg/mL of LENILUTS^®^ showed, respectively, a 1.7 ± 0.2- and 3.9 ± 0.0-fold change in caspase 3/7 activation compared to control, while a lower, yet significant, increase was also observed for CF (1.9 ± 0.1-fold change compared to control, respectively) (Figure 3A and Table S4). As shown in Figure 3B and Table S4, the same trend was maintained in the inflamed condition, even if with a milder caspase 3/7 activation (9.4 ± 1.2-, 3.6 ± 0.5- and 7.9 ± 0.6-fold change compared to inflamed, untreated in vitro prostate model for LENILUTS^®^, CF and Finasteride, respectively). As expected, the positive control of pro-apoptotic activity, staurosporine (STS), effectively induced apoptosis in both inflamed and non-inflamed conditions.

The activation of the apoptotic process in LNCaP prostate cells by LENILUTS^®^, in both inflamed and non-inflamed conditions, can be explained by the presence of curcumin among the active principles of the formulation. Indeed, curcumin and its active principles (i.e., curcuminoids), are well known to induce apoptosis in tumoral cells [37]. Similarly, the S. repens extract contained in the CF formulation is endowed with pro-apoptotic properties [38].

### 3.5. Impact of LENILUTS^®^, CF and Dutasteride Treatment on 5αR Activity in an In Vitro Prostate Model 

**Figure 4:**
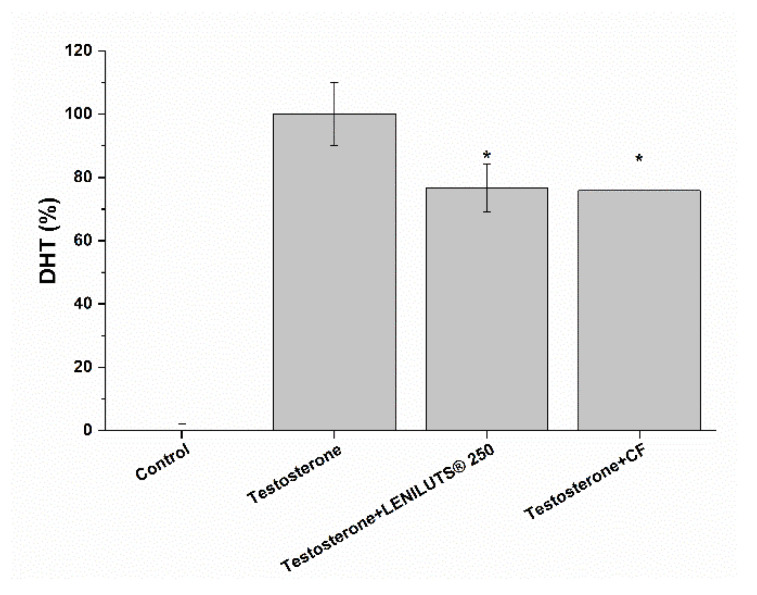
Percentage of DHT produced by 5αR from testosterone reduction, calculated compared to control, in the presence or absence of LENILUTS^®^ and CF. * *p* < 0.05 vs. testosterone.

5αR is fundamental in the insurgence and development of some prostatic pathologies (e.g., BPH). Indeed, it stimulates PSA production through dihydrotestosterone, a hormone characterized by a higher androgenic activity compared to testosterone itself [39]. As such, 5αR activity inhibition may be indicative of a positive effect at the prostate level. As shown in Figure 4 and Table S5, the addition of testosterone to 5αR, in the presence of NADPH, stimulated enzyme activity, leading to an increase in DHT synthesis compared to the control. LENILUTS^®^ was shown to be more effective at reducing DHT production than CF (with a 51.0 and 24.2% reduction for LENILUTS^®^ 250 µg/mL and CF, respectively).

### 3.6. Effect of LENILUTS^®^, CF and Dutasteride on the Release of PSA

**Figure 5:**
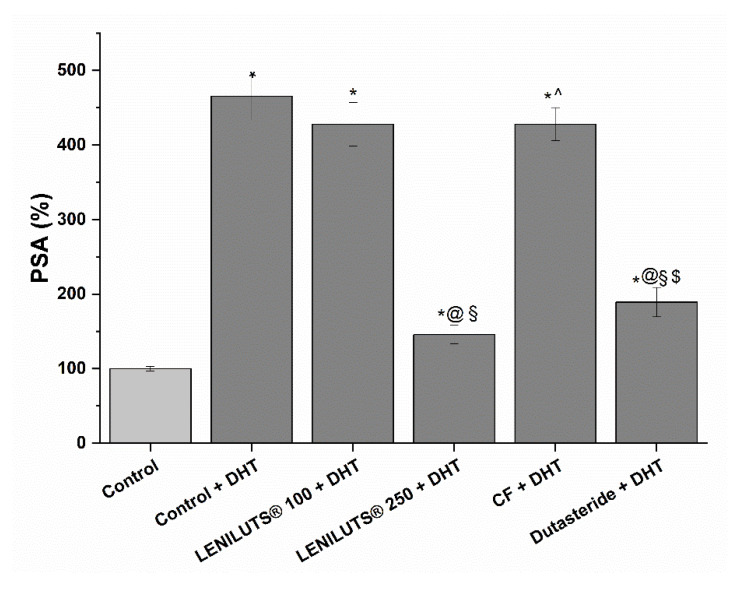
Prostate-specific antigen release in LNCaP prostatic cells following stimulation with DHT and treatment with LENILUTS^®^, CF and Dutasteride. * *p* < 0.05 vs. Control, @ *p* < 0.05 vs. Control + DHT, § *p* < 0.05 LENILUTS 100 + DHT, ^ *p* < 0.05 vs. LENILUTS 250 + DHT, $ *p* < 0.05 vs. CF + DHT. 5.

PSA is a protein released from the prostatic epithelium, and its increase in the bloodstream is associated with the development of prostatic pathologies, such as BPH. As such, PSA is a useful marker for assessing the potentially positive effect of a formulation at the prostatic level. Following stimulation with androgenic hormone DHT, a significant increase in PSA release (361.2 ± 11.9% increase compared to untreated control) was observed in the in vitro prostate model (Figure 5, Table S6). Both LENILUTS^®^ (500 µg/mL) and Dutasteride (5.9 µg/mL) significantly reduced PSA release, with LENILUTS^®^ 500 µg/mL being the most effective (145.7 ± 12.5% and 189.3 ± 19.7% PSA release, respectively, compared to 465.4 ± 31.8% PSA release for the DHT-stimulated control) (Figure 5, Table S6). Conversely, no decrease in PSA release from DHT-stimulated LNCaP cells was observed for CF. The effect of LENILUTS^®^, CF and Dutasteride on the PSA release of non-DHT-stimulated LNCaP cells is reported in Figure S3 and Table S7.

### 3.7. Bioaccessibility of LENILUTS^®^ Active Principles

The therapeutic application of active principles of plant origin, such as beta-sitosterol or curcumin, is hindered by their poor solubility in aqueous media, such as digestive fluids. Even if their solubility during the digestive process is slightly improved by the emulsifying activity of bile salts, they are far from efficient. To investigate the bioaccessibility of its active principles, we exposed a single dose of LENILUTS^®^ to an in vitro digestion procedure mimicking that in a human adult, with the aim of evaluating the total amount of active principles and the apparent bioaccessible fraction (i.e., the soluble part released from its matrix), which includes the portion that is available for absorption. As shown in Table 2, the recovery values of the active principles, calculated as the ratio between measured and expected active principle content, were 67.0%, 4.6% and 3.5% for curcumin, beta-sitosterol and OPCs, respectively, indicating that the stability of the active principles was affected by the digestive process. As a consequence of degradation and the lipophilic nature of the active principles, the bioaccessible fraction (i.e., the supernatants) of the active principles was 5.1% for curcumin (3.4 mg/dose), 17.1% for beta-sitosterol (0.7 mg/dose), and 26.6% (0.2 mg/dose) for OPCs.

### 3.8. Impact of Digested LENILUTS^®^ on Intestinal Epithelium Viability

**Figure 6:**
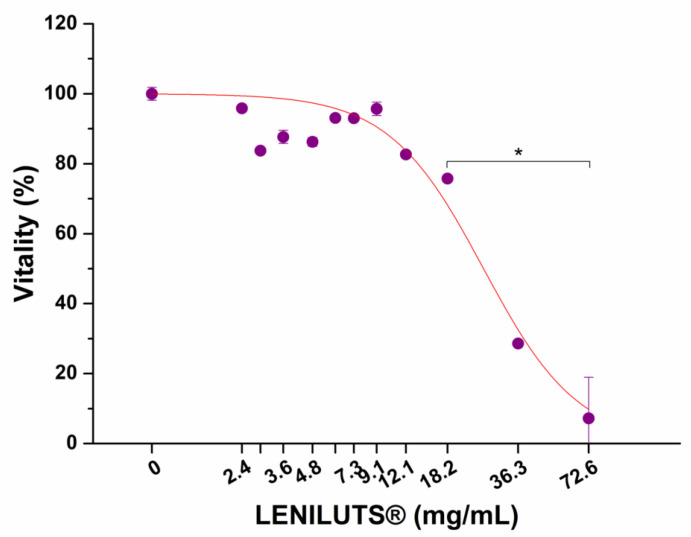
Impact of increasing concentration of digested LENILUTS^®^ on intestinal mucosa viability evaluated by MTS assay. * *p* < 0.05.

Other than demonstrating efficacy, therapeutic formulations must fulfil another requirement: that of safety. Indeed, taking into consideration their dose and posology, formulations should not negatively impact the organism. In particular, damage to the intestinal epithelium must be avoided, since this may lead to a decrease in absorption efficiency. As such, before measuring it’s the bioavailability of its active principles, the impact of digested LENILUTS^®^ on the viability and integrity of the intestinal epithelium was assessed. To this end, intestinal monolayers were exposed to increasing concentrations of the bioaccessible fraction of the formulation (i.e., the supernatant), and dose–response curves were obtained (Figure 6). As emerged from the dose–responses curves, LENILUTS^®^ showed an adverse effect on the intestinal epithelium starting at a concentration of 18.2 mg/mL. The formulation’s highest non-toxic concentration, 12.1 mg/mL, was considered for the evaluation of the bioavailability of the active principles.

### 3.9. Active Principles Bioavailability

Based on the impact of digested LENILUTS^®^ on intestinal epithelium viability and posology, we performed experiments for determining beta-sitosterol, curcuminoids and OPCs bioavailability.

#### Curcumin

In Table 3, the curcumin bioavailability data, obtained following exposure of intestinal epithelium to digested LENILUTS^®^ for 1 and 3 h, are summarized. After 1 h exposure, no absorption of curcumin was detected, while a 1.7% absorption, corresponding to 2.8 ± 0.3 ng/mL, was observed in the basolateral (serosal) compartment following 3 h treatment. Since one of the factors limiting curcumin bioavailability is its tendency to accumulate intracellularly before being released in the bloodstream [40], we measured the intracellular accumulation of curcumin at the intestinal epithelium level. As expected, a time-dependent accumulation of curcumin was observed, with a 4-fold increase from 1 to 3 h of treatment (9.8% to 32.2%, respectively) (Table 3).

The overall absorption of curcumin from the bioaccessible fraction, considering both the serosal compartment and intracellular concentration, was 1.9 ng/mL and 37.8 ng/mL after 1 and 3 h exposure, respectively (Table 3). As highlighted in Table 3, both beta-sitosterol and OPCs were degraded during the digestive process (4.6% and 3.5% recovery). However, despite its low bioaccessible fraction, absorption at the basolateral (serosal) compartment was observed for beta-sitosterol following 3 h exposure of the intestinal epithelium to the digested LENILUTS^®^ formulation (Table 4). Conversely, the absorbed concentration of OPCs was lower than the HPLC limit of detection (LOD). As a consequence, no OPCs were detected at the basolateral (serosal) compartment at either exposure time (i.e., 1 and 3 h).

### 3.10. Impact of Digested Formulations on Intestinal Mucosa Viability and Integrity

**Figure 7:**
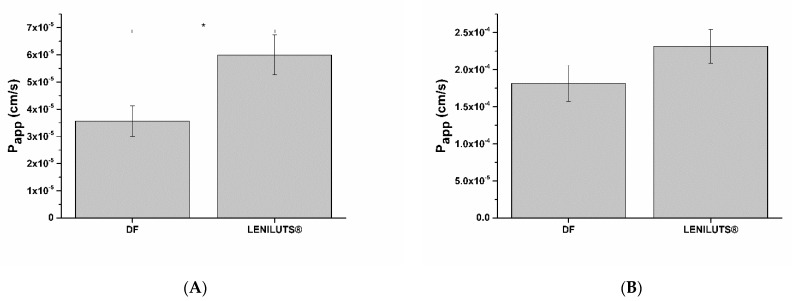
Apparent permeability (Papp) of intestinal epithelium exposed to digestive fluids (DF; control) and diluted digested formulation for 1 (**A**) and 3 h (**B**). * *p* < 0.05.

After exposure of intestinal epithelia to digested formulations, Caco-2 monolayer viability and barrier integrity were analyzed. As expected from the obtained dose–response curve, no significant reduction in viability was observed following treatment at both considered times (i.e., 1 and 3 h) with the tested formulations. Following 1 h exposure, LENILUTS^®^ slightly increased the apparent permeability of the intestinal epithelium (Papp) (Figure 7A), while no significant effect was observed after 3 h of treatment (Figure 7B).

**Figure 8:**
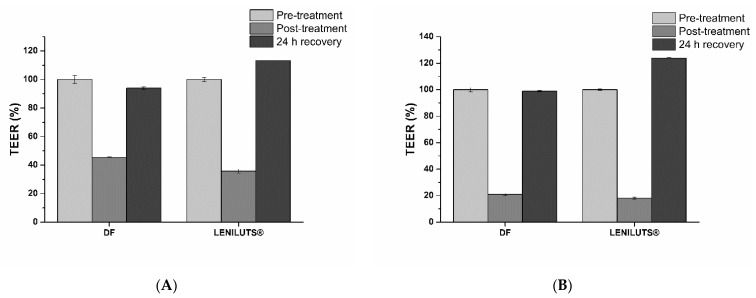
Trans-epithelial electrical resistance (TEER) trend following 1 (**A**) and 3 h (**B**) exposure to digested LENILUTS^®^.

As expected from its limited effect on the intestinal epithelium apparent permeability, digested LENILUTS^®^ only reduced TEER (trans-epithelial electrical potential) temporarily, and its values recover fully within 24 h (Figure 8).

## 4. Conclusions

Benign prostatic hyperplasia (BPH) is a term used to indicate benign growth of the prostate, and it is histologically observed on the basis of new glandular or stromal growth. BPH is also used to describe a pathological condition associated with lower urinary tract symptoms (LUTS). The incidence of BPH-associated LUTS increases with each decade of life (beyond 40 years of age), and it represents a significant burden in aging men and may impair quality of life [1]. The etiology of BPH is complex, with different underlying mechanisms, such as persistent and chronic inflammation, circulating hormonal level deregulation, aberrant wound repair processes, and steroid-mediated cell proliferation [4,7,8,9]. The use of extracts from plants and herbs for medicinal purposes (phytotherapy), including in the treatment of BPH, has been growing steadily in most countries. The most widely used phytotherapeutic agent for the treatment of BPH is the extract of the American saw palmetto or dwarf palm plant, *Serenoa repens.* Several studies have demonstrated that *Serenoa repens* exerts its biological activity through several mechanisms of action, including antiestrogenic and antiandrogenic effects, anti-inflammatory effects, and a decrease in available sex hormone-binding globulin. Despite this plethora of cellular effects, *Serenoa repens* extract is able to mitigate only a portion of BPH-related symptoms [14,15,16]. To improve BPH-related LUTS treatment, a new multi-active-principle-based formulation, LENILUTS^®^, has been proposed. Our in vitro approach demonstrated that the presence of multiple active principles improved the overall efficacy of the LENILUTS^®^ formulation by enhancing its anti-inflammatory, anti-androgenic and pro-apoptotic activities. Indeed, our results clearly show that the association of multiple active principles decreases the release of pro-inflammatory cytokine IL-1β and TNF-α more efficiently when compared to formulations based on *Serenoa repens* only. Furthermore, compared to a *Serenoa repens*-based commercial formulation, LENILUTS^®^ is more effective at reducing some BPH-connected symptoms, such as PSA release, while maintaining better safety with respect to the prostate. However, at present, the effectiveness of LENILUTS^®^ is limited by the poor bioaccessibility and bioavailability of its active principles. Consequently, further improvements in LENILUT^®^ delivery technology are needed. In conclusion, the LENILUTS^®^ formulation, once the delivery technology has been improved and perfected, might be useful in the treatment of BPH and LUTs, in particular compared to formulations based on *Serenoa repens* only.

## Figures and Tables

**Table 1:** Hydrophilic, lipophilic, and total ORAC values of the product LENILUTS^®^, expressed as micromole Trolox equivalents per gram of formula (µmol TE/g) ± standard deviation (SD).

Product	Hydrophilic ORAC (µmol TE/g ± SD)	Lipophilic ORAC (µmol TE/g ± SD)	Total ORAC (µmol TE/g ± SD)
LENILUTS^®^	32.5 ± 2.2	506.5 ± 61.2	539.0 ± 63.4

**Table 2:** Bioaccessibility of the active principles contained in the LENILUTS^®^ formulation following in vitro digestion. Data are expressed as mean ± standard deviation.

Active Principle	Recovery (%)	Supernatant (%)
CURCUMIN	67.0 ± 5.4	5.1 ± 0.1
BETA-SITOSTEROL	4.6 ± 0.1	17.1 ± 1.0
OPCs	3.5 ± 0.3	26.6 ± 7.6

**Table 3:** Curcumin bioavailability and cellular accumulation following intestinal epithelium exposure to LENILUTS^®^ for 1 and 3 h. Results are expressed as percentage of absorption and concentration (mean ± standard deviation). N.d.: not determined.

	1 h		3 h	
Curcumin	Absorption (%)	Concentration (ng/mL)	Absorption (%)	Concentration (ng/mL)
**Serosal**	n.d.	n.d.	1.7 ± 0.1	2.8 ± 0.3
**Intracellular**	9.8 ± 3.8	1.9 ± 0.7	32.2 ± 4.0	35.0 ± 3.4
**Absorbed**		1.9		37.8

**Table 4:** Beta-sitosterol bioavailability and cellular accumulation following intestinal epithelium exposure to LENILUTS^®^ for 1 and 3 h. Results are expressed as percentage of absorption and concentration (mean ± standard deviation). N.d.: not determined.

	1 h		3 h	
Beta-Sitosterol	Absorption (%)	Concentration (ng/mL)	Absorption (%)	Concentration (ng/mL)
**Serosal**	n.d.	n.d.	62.0 ± 3.1	440.8 ± 66.2

## Data Availability

The data presented in this study are available on request from the corresponding author.

## Supplementary Materials

The following supporting information can be downloaded at: https://www.mdpi.com/article/10.3390/pharmaceutics14091866/s1, Table S1. Composition of analyzed formulations. Figure S1. Absorption (A) spectrum (from 200 to 800 nm) and fluorescence spectrum (B) (excitation 420 nm; emission 450 to 800 nm) of DMSO-resuspended curcumin. Figure S2. Impact of Diclofenac on in vitro prostatic model vitality, following 6 h exposure. * *p* < 0.05. Table S2. EC50 values of LENILUTS^®^, CF and *Dutasteride* at considered exposure times. Values are reported as mean ± standard deviation. Table S3. IL-1β and TNF-α pro-inflammatory cytokines release variation in inflamed LNCaP-based in vitro prostate model following treatment with LENILUTS^®^, CF and Diclofenac, compared to the inflamed, non-treated model (Ctrl). LENILUTS^®^ is endowed with a significantly higher anti-inflammatory activity compared to CF and diclofenac (*p* < 0.05). Table S4. Change in 3/7 caspases activation compared to the control in the normal and inflamed prostate cell model, following treatment with STS (positive control), LENILUTS^®^ formula, CF and Diclofenac. Table S5. Percentage values of DHT released from LNCaP cells stimulated with testosterone, and treated with LENILUTS^®^, CF and the specific 5-α reductase inhibitor Dutasteride, compared to non-stimulated cells (Ctrl). LENILUTS^®^ 5-α reductase inhibition is significantly higher compared to CF (*p* < 0.05). Table S6. Percentage values of PSA release from DHT-stimulated LNCaP cells following treatment with LENILUTS^®^, CF and Dutasteride, compared to control (Ctrl; unstimulated cells). LENILUTS^®^ is more effective in reducing PSA production by DHT-stimulated LNCaP cell compared to CF (*p* < 0.05). Figure S3. Prostate specific antigen (PSA) release in LNCaP prostatic cells treated with LENILUTS^®^, CF and Dutasteride. LENILUTS^®^ is more effective in reducing PSA production by LNCaP cell compared to CF and Dutasteride. * *p* < 0.05. Table S7. LNCaP-released PSA percentage values following treatment with LENILUTS^®^, CF and Dutasteride, compare to untreated control (Ctrl). LENILUTS^®^ is more effective in reducing PSA production by LNCaP cell compared to CF and Dutasteride (*p* < 0.05).
