# Supplementary material for: Prostatic Therapeutic Efficacy of LENILUTS®, a Novel Formulation with Multi-Active Principles
Source: Pharmaceutics. 2022 Sep 5;14(9):1866. doi: 10.3390/pharmaceutics14091866 (PMC9506015; doi:10.3390/pharmaceutics14091866)
Supplement: Supplementary file 1 [file pharmaceutics-14-01866-s001.zip › pharmaceutics-1812811-supplementary.pdf]

Supplementary Materials

Table S1. Composition of analyzed formulations.

Formulation	Active Principle	mg/tablet
**LENILUTS®**	Pine bark (*Pinus ssp.*) e.s.<br>*min. titr. 70% beta-sitosterolo*	135<br>*94.50*
	Curcuma (*Curcuma longa* L., ryzom) e.s.<br>*titr. 95% curcuminoids*	105<br>*99.75*
	Pine bark (*Pinus massoniana Lamb.*) e.s.<br>*titr. 95% oligomeric proanthocyanidins (OPCs)*	21<br>*19.95*
**CF**	Lipo-sterolic extract of *Serenoa repens*	320 mg

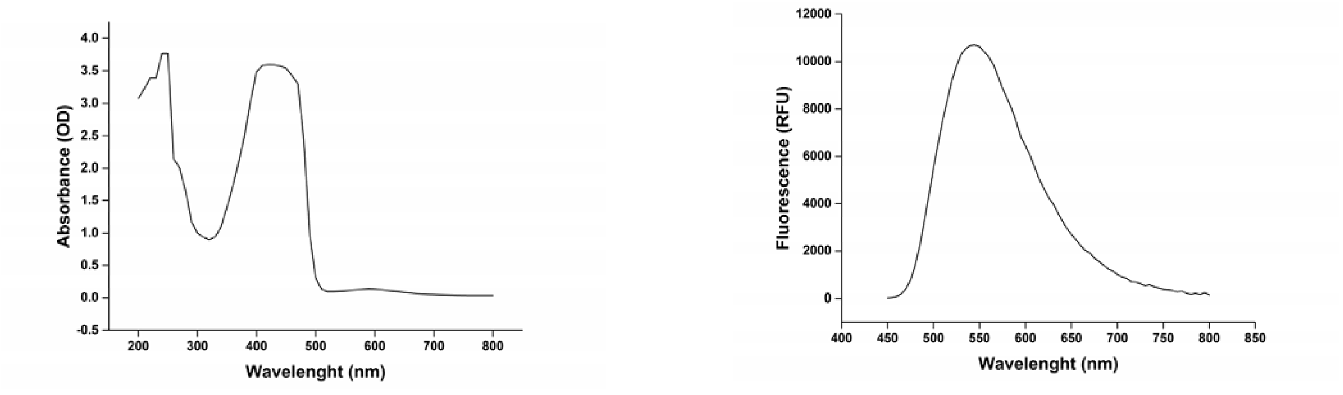

A **B**

Figure S1. Absorption (**A**) spectrum (from 200 to 800 nm) and fluorescence spectrum (**B**) (excitation 420 nm; emission 450 to 800 nm) of DMSO-resuspended curcumin.

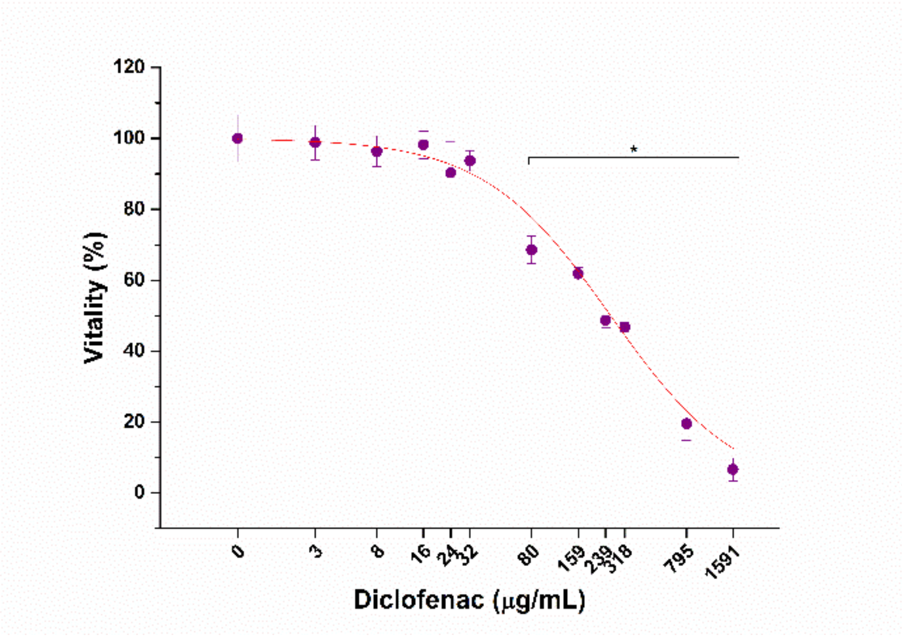

Figure S2. Impact of Diclofenac on in vitro prostatic model vitality, following 6 h exposure. * $p < 0.05$

Table S2. EC50 values of LENILUTS®, CF and *Dutasteride* at considered exposure times. Values are reported as mean ± standard deviation.

	EC50 (µg/mL)	
	**6 h**	**24 h**
**LENILUTS®**	821	654
**CF**	128	159
**Dutasteride**	> 7.93	

Table S3. IL-1β and TNF-α pro-inflammatory cytokines release variation in inflamed LNCaP-based in vitro prostate model following treatment with LENILUTS®, CF and Diclofenac, compared to the inflamed, non-treated model (Ctrl). LENILUTS® is endowed with a significantly higher anti-inflammatory activity compared to CF and diclofenac ($p < 0.05$).

	**IL-1β (fold change)**	**TNF-α (fold change)**
**Ctrl**	11.0 ± 0.0	25.3 ± 4.2
**LENILUTS® 250**	4.9 ± 0.2	18.6 ± 3.0
**LENILUTS® 500**	1.1 ± 0.1	2.3 ± 2.4
**CF**	7.7 ± 0.1	39.4 ± 5.5
**Diclofenac**	9.7 ± 0.0	38.4 ± 5.5

Table S4. Change in 3/7 caspases activation compared to the control in the normal and inflamed prostate cell model, following treatment with STS (positive control), LENILUTS® formula, CF and Diclofenac.

	3/7 caspases activation (change in activation)	
	**Normal**	**Inflamed**
**STS**	28.9 ± 1.5	22.8 ± 1.2
**Control**	1.0 ± 0.0	1.0 ± 0.2
**LENILUTS® 250**	1.7 ± 0.2	1.3 ± 0.2
**LENILUTS® 500**	3.9 ± 0.0	1.6 ± 0.3
**CF**	2.1 ± 0.4	1.6 ± 0.2
**Diclofenac**	0.8 ± 0.1	0.9 ± 0.1

Table S5. Percentage values of DHT released from LNCaP cells stimulated with testosterone, and treated with LENILUTS®, CF and the specific 5-α reductase inhibitor Dutasteride, compared to non-stimulated cells (Ctrl). LENILUTS® 5-α reductase inhibition is significantly higher compared to CF ($p < 0.05$)

	**DHT (%)**
**Ctrl**	0.0 ± 0.0
**Testosterone**	100.0 ± 10.0
**LENILUTS®**	76.7 ± 7.7
**CF**	75.8 ± 7.6

Table S6. Percentage values of PSA release from DHT-stimulated LNCaP cells following treatment with LENILUTS®, CF and Dutasteride, compared to control (Ctrl; unstimulated cells). LENILUTS® is more effective in reducing PSA production by DHT-stimulated LNCaP cell compared to CF ($p < 0.05$).

	**PSA (%)**
**Ctrl**	100.0 ± 1.6
**Ctrl + DHT**	465.4 ± 31.8
**LENILUTS® 100+ DHT**	427.8 ± 29.3
**LENILUTS® 250+ DHT**	145.7 ± 12.5
**CF + DHT**	428.1 ± 22.0
**Dutasteride® + DHT**	189.3 ± 19.7

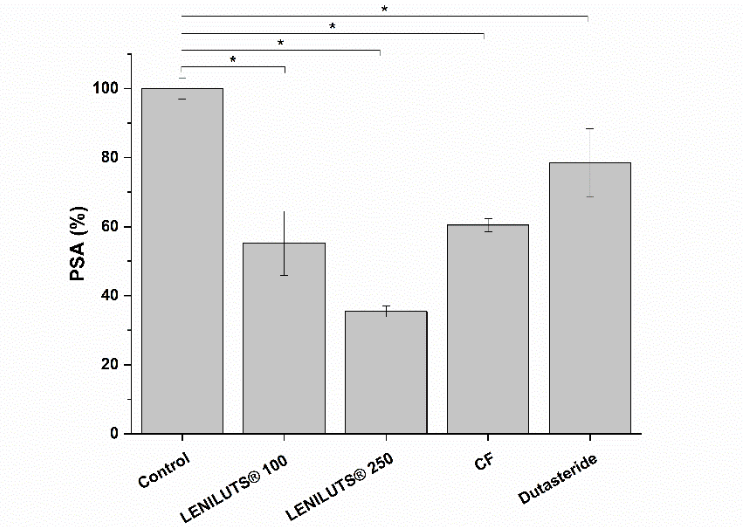

Figure S3. Prostate specific antigen (PSA) release in LNCaP prostatic cells treated with LENILUTS®, CF and Dutasteride. LENILUTS® is more effective in reducing PSA production by LNCaP cell compared to CF and Dutasteride. * $p < 0.05$.

Table S7. LNCaP-released PSA percentage values following treatment with LENILUTS®, CF and Dutasteride, compare to untreated control (Ctrl). LENILUTS® is more effective in reducing PSA production by LNCaP cell compared to CF and Dutasteride ($p < 0.05$).

	**PSA (%)**
**Ctrl**	100.0 ± 3.1
**LENILUTS® 100**	55.2 ± 9.3
**LENILUTS® 250**	35.4 ± 1.5
**CF**	60.4 ± 1.9
**Dutasteride**	78.5 ± 9.8
